# Fundamentals and future applications of electrochemical energy conversion in space

**DOI:** 10.1038/s41526-022-00242-3

**Published:** 2022-11-24

**Authors:** Katharina Brinkert, Philippe Mandin

**Affiliations:** 1 Department of Chemistry, University of Warwick, Coventry, CV4 7AL UK; 2 Center for Applied Space Technology and Microgravity (ZARM), University of Bremen, 28359 Bremen, Germany; 3 IRDL UMR CNRS 6027, Energie et Hydrogène, ENSIBS, Université de Bretagne Sud, Lorient, France

**Keywords:** Batteries, Electrochemistry, Fuel cells

## Abstract

Long-term space missions require power sources and energy storage possibilities, capable at storing and releasing energy efficiently and continuously or upon demand at a wide operating temperature range, an ultra-high vacuum environment and a significantly reduced buoyant force. Electrochemical energy conversion systems play already a major role e.g., during launch and on the *International Space Station*, and it is evident from these applications that future human space missions - particularly to Moon and Mars - will not be possible without them. Here, we will provide an overview of currently existing electrochemical conversion technologies for space applications such as battery systems and fuel cells and outline their role in materials design and fabrication as well as fuel production. The focus lies on the current operation of these energy conversion systems in space as well as the challenges posed on them by this special environment. Future experiment designs which could help elucidating and optimizing their key operating parameters for an efficient and long-term operation are discussed.

## Introduction

**Fig. 1:**
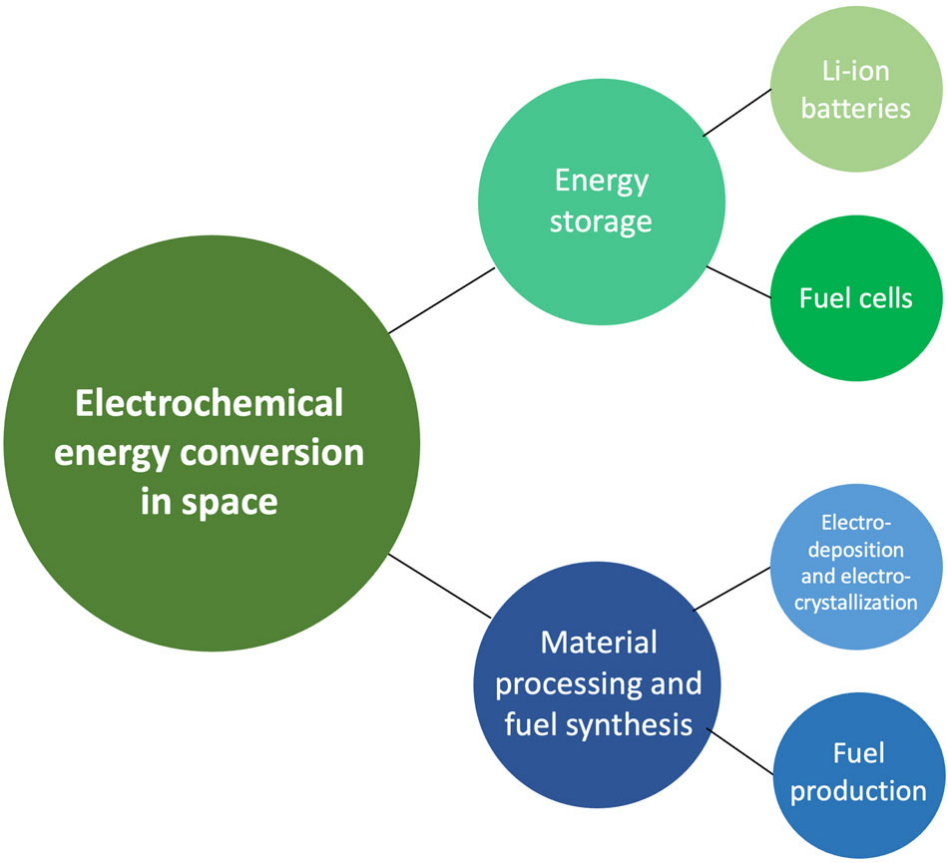
Overview of electrochemical energy conversion topics discussed in this article.

Robust electrochemical systems hosting critical applications will undoubtedly be key to the long-term viability of space operations. To the fore, electrochemistry will play an important role in energy storage and power generation, human life support, sensoring as well as in-situ resource utilization (ISRU). Of particular interest is the application of electrochemistry in energy conversion and storage as smart energy management is also a particular challenge in space^1–3^. Electrochemical systems such as batteries, fuel cells and (photo-)electrolysers are subject to extensive research efforts to meet the challenges posed by space such as an ultra-high vacuum environment, space radiation and temperatures as low as −233 °C found at the shadowed lunar polar craters^4–6^. Launches additionally cause vibrations, shocks and acceleration^4^. The near-absence of gravitation represents another obstacle as all electrochemical energy conversion systems involve fundamental processes such as chemical and/or electrochemical nucleation and the growth of crystals, thin (film) layers and gas bubbles. Here, we will provide an overview of key electrochemical energy conversion technologies which already operate in space (e.g., onboard the International Space Station, ISS) or which are currently under development for space applications. We outline their fundamental operating principles and point towards challenges or notable differences during their operation in space. As (photo-)electrolyzer systems and their applications in life support systems are already described in detail in another article in this issue – including the challenges involved with gas bubble formation and detachment in reduced gravitational environments – we will solely focus on electrochemical energy conversion in batteries and fuel cells as well as the application of electrochemistry in manufacturing processes of materials and the production of fuels in space (see overview Fig. 1). Open research questions and suggestions for future experiments are outlined.

## Electrochemical energy storage, materials processing and fuel production in space

### Batteries for space applications

The primary energy source for a spacecraft, besides propulsion, is usually provided through solar or photovoltaic panels^7^. When solar power is however intermittent, storage of energy is required in rechargeable batteries, operating in a harsh space environment which impacts their performances^8,9^. In recent years, several exploration space missions have been developed for moon and Mars, increasing the needs of batteries capable to sustain long solar eclipse periods, nighttime, yet insuring data transmission as well as powering instruments and protecting electronics from cold temperature. Interplanetary missions require rechargeable batteries which possess unique performance characteristics: they should have a high specific energy, wide operating temperatures (e.g., −233 °C to +114 °C for applications on the Moon^6^) and demonstrated safety and reliability^5^. Space applications also require batteries which can provide maximum electrical energy at minimum weight and volume. Long cycle lives (>30,000 cycles)^5^ are generally required for an orbiting spacecraft, whereas a long active shelf life is critical for planetary probes (>7–10 years)^5^. Rechargeable batteries are commonly used to provide power during (i) launch and post-launch until the deployment of solar panels becomes possible, (ii) cruise anomalies or trajectory control maneuvers of the space craft, and (iii) sun eclipse periods, to power the spacecraft, equipment and payload, (iv) nighttime or eclipse time experimentation, (v) for firing pyros and rockets for altitude control as well as (vi) communication and data transmission and (vii) to keep the electronics in a specified temperature range^5^. Batteries in planetary orbiters have the benefit of a controlled thermal environment^4,5^. Surface missions, on the other hand, require batteries that can operate in extreme environments with respect to temperature and radiation^9^. Until the late 1990s, the energy storage needs for all space missions were primarily met using aqueous rechargeable battery systems such as Ni-Cd, Ni-H_2_ and Ag-Zn and are now majorly replaced by lithium-ion batteries (LIBs)^4,5,8,9^. In the 1970s, L. Thaller at the National Aeronautics and Space Administration (NASA) also developed the so-called ‘redox flow battery’ (RFB) based on an iron-chromium (Fe/Cr) redox couple^10^. This system replaces one or two heterogeneous electrochemical reactions with the transformation of homogeneous electroactive species. Two soluble redox couples contained in external electrolyte tanks sized according to their application are supplied to flow-through electrodes where chemical energy is converted to electrical energy (discharge) or vice versa (charge). The anode and cathode compartments are separated by a membrane (or separator) which selectively allows cross-transport of non-active species (such as H^+^, Cl^-^) for maintaining electrolyte neutrality^11^. RFBs are more like regenerative fuel cells (discussed in another article in this issue in more detail) and power and energy capacity can be designed separately. The power (kW) is determined by the size of the electrodes and the number of cells in the stack. The concentration and volume of the electrolyte determine the energy storage capacity. A major issue in dealing with RFBs are the shunt or parasitic currents which lead to self-discharge and energy loss^11,12^. This current loss occurs because the anode and cathode sides of the cells are fed with pumped electrolyte in parallel. The voltage difference over different cells creates the shunt current that flows through the conductive electrolyte^11^. Optimum designs are therefore critical to minimize the shunt current and improve other performance parameters.

LIBs are numerous and provide the largest number of energy storage devices in terms of power (W) and stored energy (kWh). In the following, we outline the pertinent, efficient, and challenging nexus between terrestrial operation principles and device requirements for space applications.

**Fig. 2:**
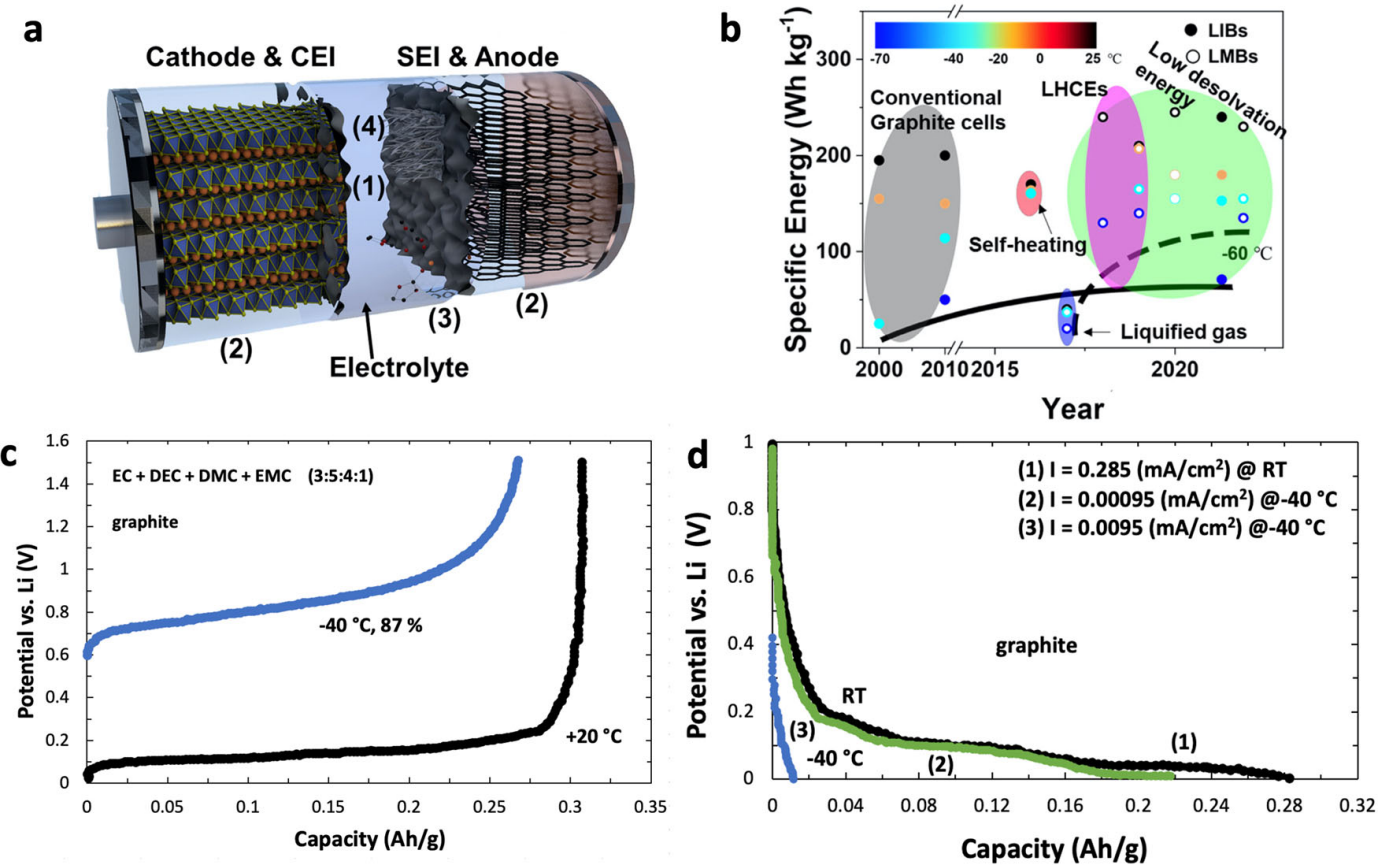
Scheme of a typical lithium-ion battery (LIB) and characteristics of operation at low and high temperatures. **a** Scheme of a typical lithium-ion battery (LIB) consisting of a layered oxide cathode, a graphite anode and the electrolyte. The numbers represent the limiting factors during low-temperature operation: (1) decline of wettability and ion conductivity of the liquid electrolyte; (2) increase of intrinsic grain-boundary resistance and sluggish Li^+^ diffusion; (3) difficult Li^+^ desolvation and slow transport through SEI (solid electrolyte interface); (4) severe Li plating on the anode. CEI stands for cathode-electrolyte interface. **b** Comparison of the energy/power density of typical commercial batteries operated at 25 °C, −20 °C and −30 °C. LMBs are high-energy lithium metal batteries and LHCE stands for localized high-concentration electrolytes. **c**, **d** Comparison of the charge (**c**) and discharge (**d**) profiles of a Li||graphite half-cell running at room temperature (RT) and −40 °C. The inset image in (**c**) is a schematic diagram of the Li^+^ diffusion during Li (de)intercalation. The inset image in **d** is the performance comparison of coke with various sizes at various temperatures (20 °C, −20 °C, −30 °C). Figure 1a, b are reprinted from Zhang et al. Critical review on low-temperature Li-ion/ metal batteries. Advanced Materials, 2022, 34, 2107899-2107930^8^. Copyright Wiley-VCH GmbH. Reproduced with permission. Figure 1c, d are redrawn from Huang et al. (2000)^14^.

The first LIB module operating stably at 104 Wh kg^−1^ for more than 1000 cycles was launched in October 2001 on the European spacecraft ‘Proba-1’^13^. A major obstacle with LIBs is however the operation at low temperatures (see Fig. 2a): (1) the liquid electrolyte suffers from a wettability and ion conductivity decrease due to an increased viscosity and/or solidification which blocks the ion transport in the electrolyte, (2) the increase of intrinsic grain-boundary resistance and sluggish Li^+^ diffusion within the electrodes suppress the (de)lithiation reactions, (3) the Li^+^ desolvation and charge transfer becomes difficult and a slow transport through the solid electrolyte interface (SEI) together with the large charge transfer resistance reduces the battery kinetics, and (4) severe Li plating on the anode under low-temperature gives rise to safety concerns^5,8,9^. Figure 2b summarizes the developments in the field of low-temperature LIBs and LMBs (lithium metal batteries) with respect to the energy density^8^.

Novel electrolytes catering to the needs of low-temperature environments are a prerequisite for cost-efficient and safe operation of LIBs in space. Generally, additives such as acylic carbonate or carboxylic esters such as dimethyl carbonate (DMC), ethyl methyl carbonate (EMC) and diethyl carbonate (DEC) are extensively researched as they reduce the viscosity of electrolytes and improve the overall ion transport kinetics in batteries^8,13^. Besides obstacles with the electrolyte, kinetic issues in the interior and/or the surface of electrodes are likely about to turn up during operation at low temperature^13,14^ (Fig. 2c, d): Huang et al. (2000) showed that the lithiation process of graphite is relatively difficult at −40 °C even at low current densities (5.4% and 42.3% of discharge capacity retention for graphite and coke at 0.0095 mA cm^−2^, respectively). The delithiation process is achievable, however, at 87 and 86% of charge capacity retention for graphite and coke, respectively^13^. Besides the diffusion problem within the graphite anode^15^, the high interface resistance linked to Li^+^ desolvation and migration through the SEI restrict the cell reversibility at low temperatures^8^. Furthermore, the high tendency of Li plating and dendrite formation is another issue when LIBs are operated at low temperatures or high rates. The potential for Li^+^ intercalation into graphite is close to that for lithium plating (within 100 mV *vs* the Li/Li^+^ redox couple)^8^ and once the overpotential of LIBs is larger than this potential gap, the Li^+^ ions are reduced on the surface of graphite rather than intercalated into its layers due to the lower nucleation barrier. The plated Li induces consecutive parasitic reactions such as the generation of a thicker SEI and ‘dead’ Li. One possibility to suppress Li plating is the mild oxidation of graphite:^8^ after thermal treatment or oxidation with concentrated nitric acid solution, graphite usually exhibits a better cycling performance at lower temperatures, caused by the reduction of unsaturated carbon atoms at the edge planes, the decline of particle size, the creation of nanovoids, nanochannels and the formation of a chemically bonded SEI.

When LIBs are however operated at optimal temperatures, a high number of charging and discharging cycles is already achievable: the LIB cells onboard the satellite REIMEI achieved 55,000 charging and discharging cycles in a controlled temperature environment of 19–22 °C^16^.

Future space exploration activities require batteries that can operate at ultra-low temperatures (probes, landers, rovers, and penetrators), withstand ultra-high G forces up to 80,000G (penetrators) and provide exceptionally long cycle life capabilities (orbiters). Furthermore, they need to be lightweight and compact. Micro- and nanorovers are considered for exploratory purposes which in turn require microbatteries and batteries on chip systems, micro-capacitors and fuel cells operating in the mW range. Earth-orbiting missions require however large-size batteries with long life cycles (e.g., in Low Earth Orbit, LEO > 50,000 at 50% depth of discharge, DOD). Future Group on Earth Observations (GEO) will however require large size batteries which can operate for 15–20 years.

### Fuel cells

**Fig. 3:**
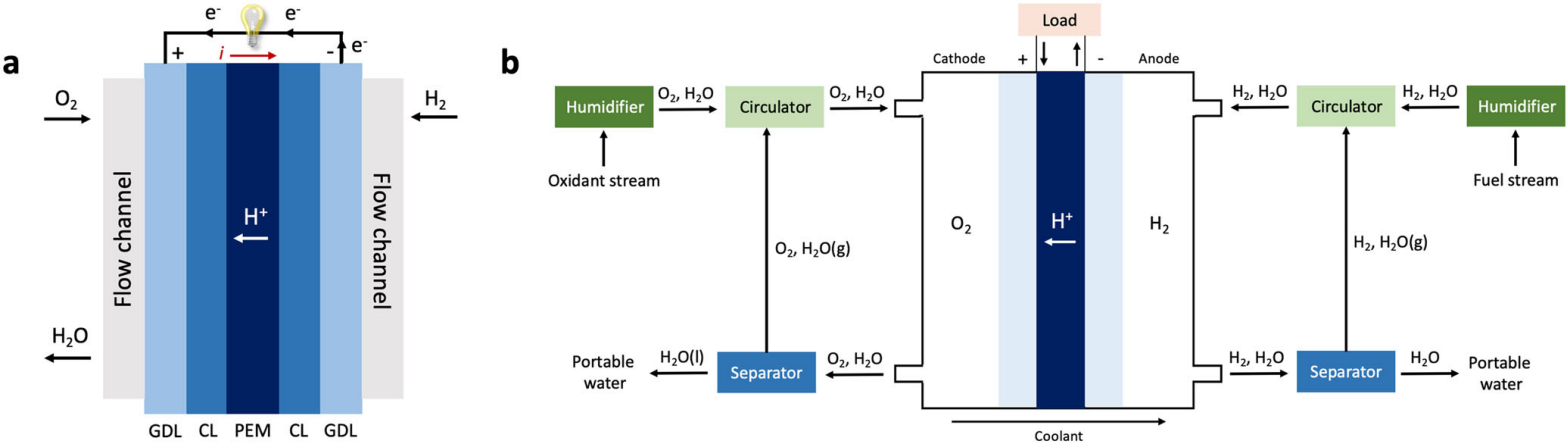
Scheme of a PEM-based fuel cell. **a** Scheme of a PEM (proton exchange membrane)-based fuel cell. GDL is the gas diffusion layer and CL the catalyst layer. **b** Extended scheme of a conventional PEMFC with an active fluid supplement system, comprising the fuel cell, gas humidifiers, an active circulation, and phase separation subsystem. Redrawn from Guo et al. (2017)^3^.

The first use of a fuel cell device in space was part of the Gemini program in August 1962^16^. Two types of hydrogen/ oxygen fuel cells have successfully been utilized to provide electric energy and potable water for several human-rated space missions: alkaline fuel cells (AFCs) have generally been more successful than ion electrolyte membrane fuel cells (IEMFCs) which utilize sulfonated polystyrene^3^. One major drawback of the IEMFCs was the high ohmic resistance of the ion electrolyte membrane which resulted in a lower operating performance^3^. With the development of a new type of thin proton exchange membrane by DuPont, Nafion, a new type of proton exchange membrane fuel cells (PEMFCs) could be designed. In the 1990s, NASA aimed at replacing the costly AFC power plants with PEMFC systems. Since then, PEMFCs are recognized as the main space fuel cell power plants for future lunar and Mars missions, reusable launch vehicles space station energy storage and portable applications^3,17,18^. The enormous life-cycle cost caused by corrosion in the AFC system is not a problem for space PEMFC devices. A first test certified that hydrogen-oxygen-based PEMFCs enable power densities >500 W kg^−1^ and 200 W L^−1^ as well as energy densities of >500 Wh kg^−1^ and 400 Wh L^−1^ (ref. ^3^). PEM-based fuel cells operate in the reverse mode of PEM electrolyzers, consisting of hydrogen and oxygen gas flow channels, gas diffusion layers (GDLs), catalyst layers (CLs) and a proton exchange membrane (PEM, Fig. 3a)^3^. They convert chemical energy stored in hydrogen into electrical energy and generate water as a byproduct and waste heat. Technologies are required which manage the complicated gas-liquid two-phase fluid and waste thermal inside the energy conversion systems to operate efficiently in a low gravity environment. Sufficient water management is required to maintain good ion migration and reactant mass transfer in PEMFCs, prerequisite for a long operating lifespan and device stability. The management difficulty is attributed to the water balance between membrane hydration and water removal. On the one hand, pre-humidified reactants that carry external water are supplied to fuel cells for sufficient PEM hydration (see representative PEMFC scheme in Fig. 3b)^3^. This is necessary to enable hydrogen cation migration from anode to cathode. On the other hand, water generated by the electrochemical reactions is expected to be timely removed. Investigations are required to further understand the mechanisms of the water discharged from the flow field in microgravity environments^19,20^. So far, only a few studies have been devoted to investigating product removal from the flow channels of the PEMFCs in short-term microgravity experiments. In reduced gravitational environments, the pressure difference between the inlet and outlet of the flow channel is the main driving force that supports water discharge away from the fuel cell. This water removal process shows a complex gas liquid two-phase flow phenomenon inside the flow field caused by the near-absence of gravity, strongly affecting the overall cell behavior. Further effort is required to control the operating conditions and improving the cell configurations to promote water removal in PEMFCs during long-term microgravity exposure. NASA has also investigated non-flow through PEMFCs, where the product water wicks through a support structure across an adjacent gas cavity through a hydrophilic membrane and into a water cavity within each cell stack^21^. There are no recirculating reactants and therefore no requirements for providing either recirculation or product water separation from two-phase reactant streams. A back-pressure regulator is however needed to maintain the adequate differential pressure between oxygen and water/coolant activities. The thinner cell stacks could have a major weight and volume advantage in comparison to flow-through systems, however the technological readiness level is far behind the flow-through PEMFCs^21^.

A large effort is also dedicated to the development of High Temperature Fuel Cells (HTFC)^22^ such as high temperature proton exchange membrane fuel cells (HT-PEMFCs) which provide advantages such as fast reaction kinetics and thus a high energy efficiency, a high tolerance to fuel/air impurities, a simple plate design and a better heat and water management. HTFCs convert the chemical energy of a fuel directly into electricity and heat and can use substrates such as coal, natural gas and biomass in combination with oxidants. According to the formation of oxygen anions, which are transported through the electrolyte from cathode to anode, three HTFC types can be distinguished: molten carbonate fuel cells (MCFCs), solid oxide fuel cells (SOFCs) and direct carbon fuel cells (DCFCs)^23^. One of the most prominent examples of applying SOFCs in space environments is the Mars Oxygen In Situ Resource Utilization Experiment (MOXIE), an oxygen-producing device located at the front of the 2020 *Perseverance* Mars Rover. It collects Martian atmosphere, containing ~96% CO_2_, and draws it through a filter to a compressor inlet^24^. The CO_2_ is then pre-heated and flows back into a stack of SOFCs, where 30–50% of the hot CO_2_ is converted to CO and O_2_. Scandia-stabilized zirconia (ScSZ) is used as an electrolyte and O^2−^ ions transport the current from cathode to anode. Due to the poor ion conductivity of the electrolyte at low temperatures, SOFCs typically operate in the 800–1000 °C range^23^. An interesting alternative are so-called ‘proton conducting electrochemical cells’ and ‘protonic fuel cells’ (PCFCs) which can overcome some of the SOFCs limitations such as detrimental anode oxidation and the low utilization efficiency of hydrocarbon fuels^25^. The significantly higher ionic conductivity and low activation energy required for proton conduction compared to the O^2−^ ion conduction in SOFCs can facilitate high performances. For example, the most recent proton conducting oxide BaZr_0.4_Ce_0.4_Y_0.1_Yb_0.1_O_3-δ_ (BZCYYb) exhibits an ion conductivity of 2.8 × 10^−2 ^S cm^−1^ at 600 °C which is about 12 times greater than the one of Y_0.08_Zr_0.92_O_2-δ_ (2.2 × 10^−3 ^S cm^−1^ at 600 °C) used in SOFCs^25^. Furthermore, the lower activation energy of BZCYYb provides the opportunity to achieve higher electrochemical performances of PCFCs with an operation temperature below 600°C, avoiding material degradation and high system costs. This makes them particularly attractive as well for electrosynthesis applications e.g., for the production of hydrogen, hydrocarbon-based fuels or ammonia.

Requirements such as a compact system design, easy re-fueling, low fuel cost, quick start-up as well as ambient temperature and pressure operation have made direct methanol fuel cells (DMFCs) interesting to power small, unaided devices in spacecrafts^26–28^. In DMFCs, methanol is directly oxidized at the anode without any reforming processes, while oxygen is introduced into the cathode for reduction^26^:

1 $${{{\mathrm{Anode}}:2{\mathrm{CH}}}}_{{{\mathrm{3}}}}{{{\mathrm{OH}} + {\mathrm{2H}}}}_{{{\mathrm{2}}}}{{{\mathrm{O}}}} \to {{{\mathrm{2CO}}}}_{{{\mathrm{2}}}} + 12{{{\mathrm{H}}}}^{{{\mathrm{ + }}}} + 12{{{\mathrm{e}}}}^{{{\mathrm{ - }}}}$$

2 $${{{\mathrm{Cathode}}:3{\mathrm{O}}}}_{{{\mathrm{2}}}} + 12{\mathrm{H}}^{{{\mathrm{ + }}}} + 12{\mathrm{e}}^{{{\mathrm{ - }}}} \to 6{{{\mathrm{H}}}}_{2}{{{\mathrm{O}}}}$$

3 $${{{\mathrm{Overall}}}}\,{{{\mathrm{cell}}}}\,{{{\mathrm{reaction}}:2{\mathrm{CH}}}}_{3}{{{\mathrm{OH}} + 3{\mathrm{O}}}}_{2} \to 2{{{\mathrm{CO}}}}_{2} + 4{{{\mathrm{H}}}}_{2}{{{\mathrm{O}}}}$$

DMFCs are studied reasonably well in terrestrial environments, however, only a few studies report investigations in microgravity. Terrestrially, the CO_2_ gas bubbles are separated from the gas diffusion layer and rise to the top of the flow channels due to buoyancy. They are then merged and pushed out of the fuel cell by the pressure difference between the inlet and outlet of the reactant streams. Anode in-situ optical images show that the detachment and rising speed of the CO_2_ gas bubbles slow down significantly in microgravity^26^. The separated diameter of carbon dioxide bubbles increase with the operation duration to the point that the cross section of the flow channels is occupied with large gas bubbles, blocking the flow channel. The CO_2_ bubble flow turns into a slug flow in microgravity, which hinders mass transfer of the methanol from the flow channel to the catalyst layer^26^. When operated at high current density, the cell performance is thus dominated by concentration polarization. An increase in the methanol feeding molarity has been found to be conducive to weaken the effect of gravitation on the phase separation obstacle^26^. Furthermore, an increase in the feeding flow rate of the methanol solution can reduce the size of CO_2_ gas bubbles and thus also improve the DMFC performance. Generally, DMFCs are able to achieve stable cell performances in short-term microgravity at low current densities. This is caused by the impact of the activation polarization on the cell performance, which is more severe than that of the concentration polarization at low current density. The effect of low gravitation on the cell performance is therefore lower than at high current density. Further studies of DMFCs in microgravity environment are required to better understand and optimize the operating conditions as well as cell configurations for space applications.

Besides hydrogen and methanol, fuel cells have been proposed utilizing ammonia as a fuel^29–31^. Ammonia has recently been considered as the main substitution for hydrogen and the next generation fuel^32^ due to its high energy density (12.6 MJ L^−1^) and the easiness of storage and transportation^29^. The electrochemical oxidation of ammonia is a mass transfer-controlled reaction and the effects of linear polarization have been investigated in microgravity environment. On Pt surfaces, the electrochemical ammonia oxidation proceeds after the widely accepted Gerischer and Maurer mechanism^29^. It can be summarized by the following equation:

4 $$2{{{\mathrm{NH}}}}_{3}\left( {{{{\mathrm{aq}}}}} \right) + 6{{{\mathrm{OH}}}}^{ - } \to {{{\mathrm{N}}}}_{2}\left( {{{\mathrm{g}}}} \right) + 6{{{\mathrm{H}}}}_{2}{{{\mathrm{O}} + 6{\mathrm{e}}}}^{ - }$$

**Fig. 4:**
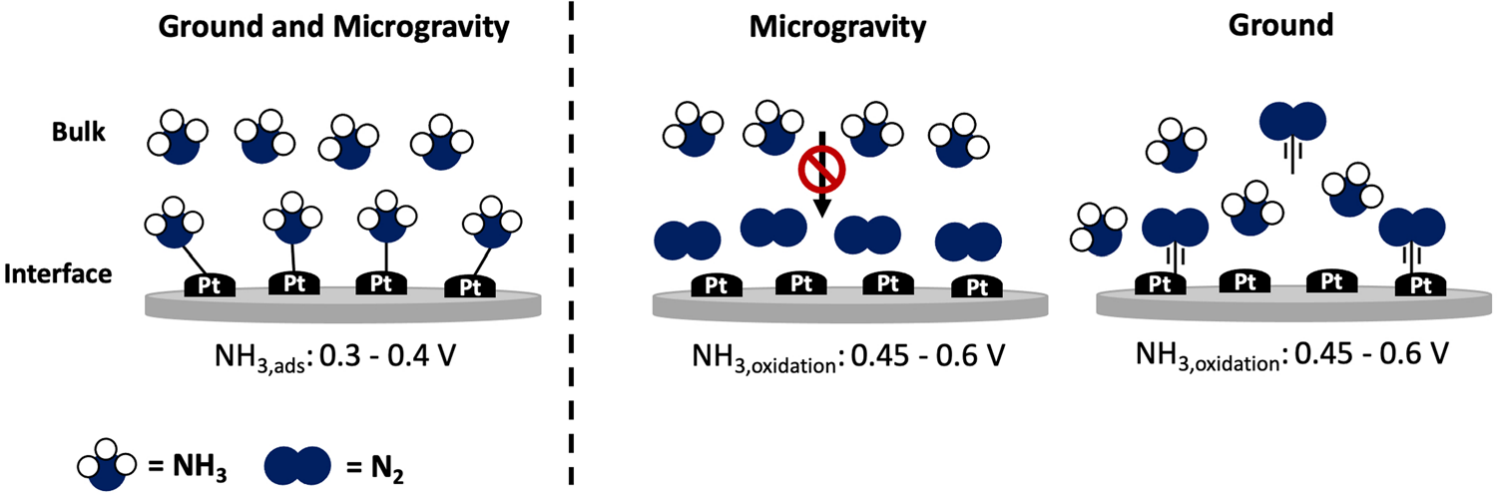
Schematic illustration of the effect of microgravity on the electrochemical ammonia oxidation reaction. The absence of macroconvectional processes hinders the removal of N_2_(g) from the electrode surface and limits the interaction of ammonia with the Pt electrocatalyst. Redrawn from Nicolau et al. (2012)^29^.

The reaction takes place at potentials between ca. 0.45 – 0.6 V vs. RHE (Reversible Hydrogen Electrode). Nicolau et al.^29^ reported that the oxidation of ammonia with different Pt-based nanocatalysts resulted in a decreased performance of 20–65% in microgravity environments in comparison to terrestrial control experiments. Similarly, Acevedo et al.^31^ showed that the performance of ammonia-based alkaline fuel cells decreased in microgravity environment. Nicolau et al. (2012) attributed the current decrease to the lack of buoyancy-driven mixing in the near-absence of gravitation (Fig. 4). The authors hypothesized that ammonia molecules might not be able to reach the electrode surface at the same rate as terrestrially possible. They observed that at ca. 0.7 V *vs*. RHE, the formation of N_2_(g) creates a stagnant gas-surface interaction that hinders ammonia molecules from interacting with the Pt surface. This leads to a significant peak current decrease compared to ground-based experiments^29^. The result was supported by Poventud-Estrada et al., who found that the morphology of the electrocatalyst itself had a significant impact on the efficiency of the reaction in microgravity: using Pt-supported mesoporous carbon electrodes with three different pore diameters, the authors showed that all three catalysts yielded in a 25–63% decrease of in ammonia oxidation current in comparison to ground-based tests within time scales of 1 s to 15 s, although a catalyst with a 137 Å-size porous nanostructure only showed a decrease of 25–48%^30^. This suggests that a careful evaluation and optimization of the electrocatalyst design, gas product removal as well as a further understanding of the governing mass transfer processes in microgravity environment are necessary to advance the development of (alternative) fuel cell concepts for space applications.

### Material processing

Besides applications in energy conversion and storage, electrochemistry can also play a vital role in low-energy, ambient temperature manufacturing processes of materials. For instance, electrochemical microfabrication offers some unique advantages for the manufacturing of advanced microelectronic components, thin-film magnet heads and micro-electro-mechanical systems which could be highly relevant for device fabrications onboard spacecrafts and in space habitats. Electrochemical microfabrication includes anodic dissolution phenomena such as electropolishing, electrochemical micromachining and furthermore, cathodic processes such as electrodeposition of thin films and multilayers as well as electrical contacts of bump and soldering alloys^33,34^. Metal electrodeposition and electrocrystallization studies have been carried out to some degree in microgravity environments as macroconvectional processes and thermal convection influencing the morphology of deposits and crystals are nearly absent in reduced gravitational environments^35–37^.

### Metal electrodeposition and electrocrystallization in microgravity

**Fig. 5:**
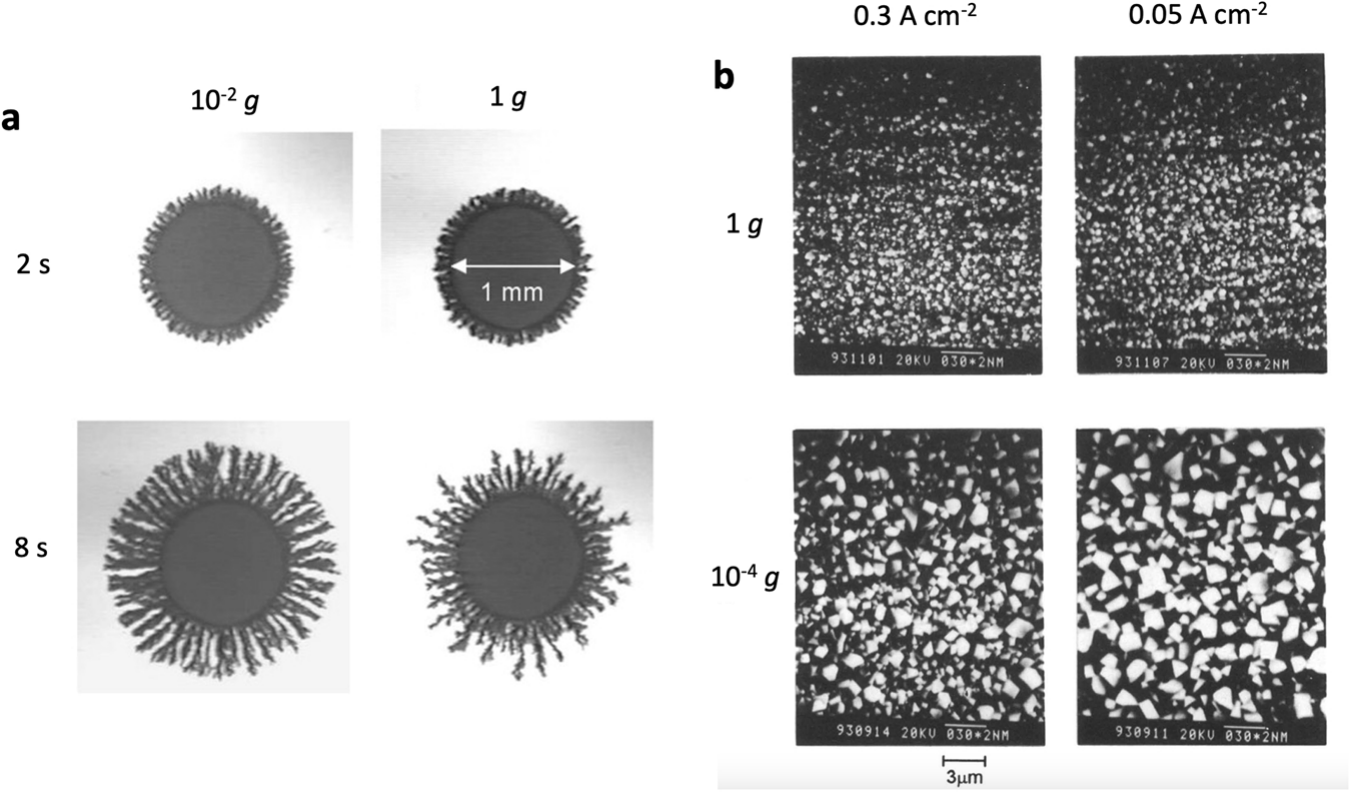
Morphological variations of electrodeposited Cu in microgravity (10^−2 ^*g*) and terrestrial experiments. (**a**) For both experiments, the current density was set to 2.5 A cm^−2^. Reprinted from Electrochim. Acta, 100, Nishikawa, K., Fukunaka, Y., Chassaing, E. & Rosso, M. Electrodeposition of metals in microgravity conditions, 342–349, 2013, with permission from Elsevier^36^. **b** Comparison of electrochemically deposited Cu during 8 s in terrestrial (1 g) and microgravity environments (10^-4 ^g) at the indicated constant current densities. Reprinted from J. Electrochem. Soc., 145, Fukunaka, H., Okano, K., Tomii, Y. & Asaki, Z. Electrodeposition of copper under microgravity conditions, 1876–1881, 1998, with permission from Elsevier^35^.

Metal electrodeposition produces a variety of deposits on substrates, including dendrites as evident in Li^+^ ion batteries. Dendrite formation leads to a short-circuit of the battery which represents a major safety issue. Hence, understanding and controlling the development of morphological metal variations during electrocrystallization and electrodeposition e.g., in LIBs, but also during electrochemical thin film deposition, is crucial in microgravity environment. It has recently been shown that even short microgravity experiments could provide information about the preliminary stages of dendritic growth in electrodeposition^35–37^. Fukunaka et al.^35^ carried out the first experiments on copper electrodeposition from an aqueous CuSO_4_ solution in a drop shaft with a microgravity duration of >8 s, applying a constant current density (Fig. 5a)^35^. Fewer, larger Cu grains with a preferential growth of lower indexes were obtained in microgravity environment (Fig. 5b)^36^. Additionally, it was observed that the electrode surface concentration of Cu^2+^ ions was lowered more quickly^35^. This was hypothesized to lead to a lower three-dimensional nucleation rate and fewer Cu nuclei on the electrode surface. When the same number of charges passes through the cathode, more coulombic charge could be distributed on one single grain, leading to the growth of larger-sized grains. At the same time, however, it was observed that the electrical conductivity was reduced with the lowered Cu^2+^ concentration near the electrode surface^35^. This was speculated to potentially influence the ionic mass transfer rate due to the migration effect. The experiment shows clearly that buoyancy-driven convection not only significantly changes concentration profiles in the electrolyte, but it also alters the morphology of the deposit. Further measurements of the Cu^2+^ ion concentration profile in proximity of the electrode surface is necessary to fully understand the morphological differences obtained during electrodeposition in microgravity environment. It has also been found that due to the time constraints, drop shaft experiments and parabolic flights could only provide insights into the initial stages of electrodeposition. In order to fully examine e.g., the deposition of lithium from ionic liquids relevant to investigate short-cuts in battery life cycles, longer microgravity times (Sounding Rocket experiments, ISS) are required.

### Electrochemistry for fuel production

Fuels - together with a liquid oxidizer such as liquid oxygen (LOX) or nitric acid - can be used in propellants as the chemical energy sources of rocket engines or in space habitats to power e.g., vehicles^32^. Most commonly, hydrocarbons or hydrogen are employed as fuels. Besides gasoline, kerosene, diesel oil and turbojet fuel, methane is currently investigated. Methane is a cryogenic fuel which is denser than liquid hydrogen and low in cost. It is particularly interesting for Mars applications as the Martian atmosphere consists of about 96% CO_2_^38^. Moreover, liquid hydrogen is used as it is the lightest and coldest, possessing a specific gravity of 0.07^32^. In combination with oxygen, it has successfully been employed as a propellant due to the high specific impulse generated upon mixing. Terrestrially, the development of sustainable energy conversion processes replacing CO_2_ by water through the hydrogen vector is a challenge which needs to be overcome within the frame of climate change. Space exploration faces similar challenges in terms of the sustainable and reliable production of fuels. Water electrolysis and fuel cells are crucial technologies which are currently investigated within this framework, their large-scale deployment requires however a reduction in cost and an increase in performance. Several new possibilities have been explored in the past to electrochemically produce fuels in space habitats on Moon and Mars and only a few, new key concepts are summarized in the following. As the main operation principle of (photo-)electrolyzer systems is outlined in a separate article in this issue, we will focus here on the application of these technologies for space exploration.

The production of hydrogen via water (photo-)electrolysis includes two coupled processes: the oxygen evolution reaction (OER)^39–47^ and the hydrogen evolution reaction (HER)^48^. The OER process plays a determining role due to its sluggish reaction kinetics. The overpotential necessary to drive the OER is higher than that for the HER, since the OER includes a four-electron/four‐ proton exchange process to produce one oxygen molecule. Towards this end, enormous efforts have been devoted to exploring and developing new, efficient and sustainable electrocatalysts for OER. Besides traditional proton exchange membrane water electrolysis (PEMWE) systems, anion exchange membrane water electrolysis (AEMWE)^49,50^ has become an attractive alternative for hydrogen production in terrestrial systems, allowing the replacement of Ir anodes by more abundant, platinum-group free transition metal oxides (TMO) and the use of less expensive anode flow fields and bipolar plates. Despite numerous publications devoted to the investigation of TMOs during the oxygen evolution reaction, the nature of the active sites and the evolution of the surface composition during the course of electrolysis is not fully understood yet. Generally speaking, the electrochemical water-splitting performance of AEMWEs is still lower than the one of PEMWEs^51–53^. The major voltage losses of a typical AEMWE are due to (1) the ohmic loss caused by the ion conduction through the membrane and (2) the activation loss consisting of both anode and cathode overpotentials due to the operation in alkaline environments. Nevertheless, AEMWEs comprising of Ni-based catalysts are already able to produce hydrogen with ∼1.5 A cm^−2^ at 1.9 V in 1 M KOH, 70°C^54^, approaching the performance of conventional PEMWEs under ambient pressure. Further investigations for optimization are however required, comprising e.g., the clarification of the role of Fe impurities in the KOH electrolyte for the OER^41,55^. It has been proposed that Fe impurities could affect the electronic structure of the Ni-based electrocatalyst, the hybridization of the Ni-O by incorporation into the Ni surface or that it could work as an OER mediator between the catalyst surface and the bulk electrolyte^41,55^. Generally, the application of AEMWEs could be interesting for space habitats where Ni is an abundant resource; this is the case on both, Moon and Mars.

One route towards the generation of green fuels and chemicals is the (photo-)electrochemical reduction of CO_2_ towards carbon-containing chemical building blocks, such as CO, methane (CH_4_) or ethylene (C_2_H_4_) but also formic acid (HCOOH) and methanol (CH_3_OH)^55–58^. Due to the kinetic stability and thermodynamic inertness of CO_2_, the choice of electrocatalyst for the reaction is important for the reactions’ efficiency and product selectivity. In the direct electrochemical scheme, the cathodic CO_2_ electroreduction is coupled with a corresponding half-cell reaction providing the requisite protons and electrons. The electrosynthesis of methane occurs according to the following equation:

5 $${{{\mathrm{CO}}}}_{{{\mathrm{2}}}}{{{\mathrm{ + 8H}}}}^{{{\mathrm{ + }}}}{{{\mathrm{ + 8e}}}}^{{{\mathrm{ - }}}} \to {{{\mathrm{CH}}}}_{{{\mathrm{4}}}}{{{\mathrm{ + 2H}}}}_{{{\mathrm{2}}}}{{{\mathrm{O}}}}$$

**Fig. 6:**
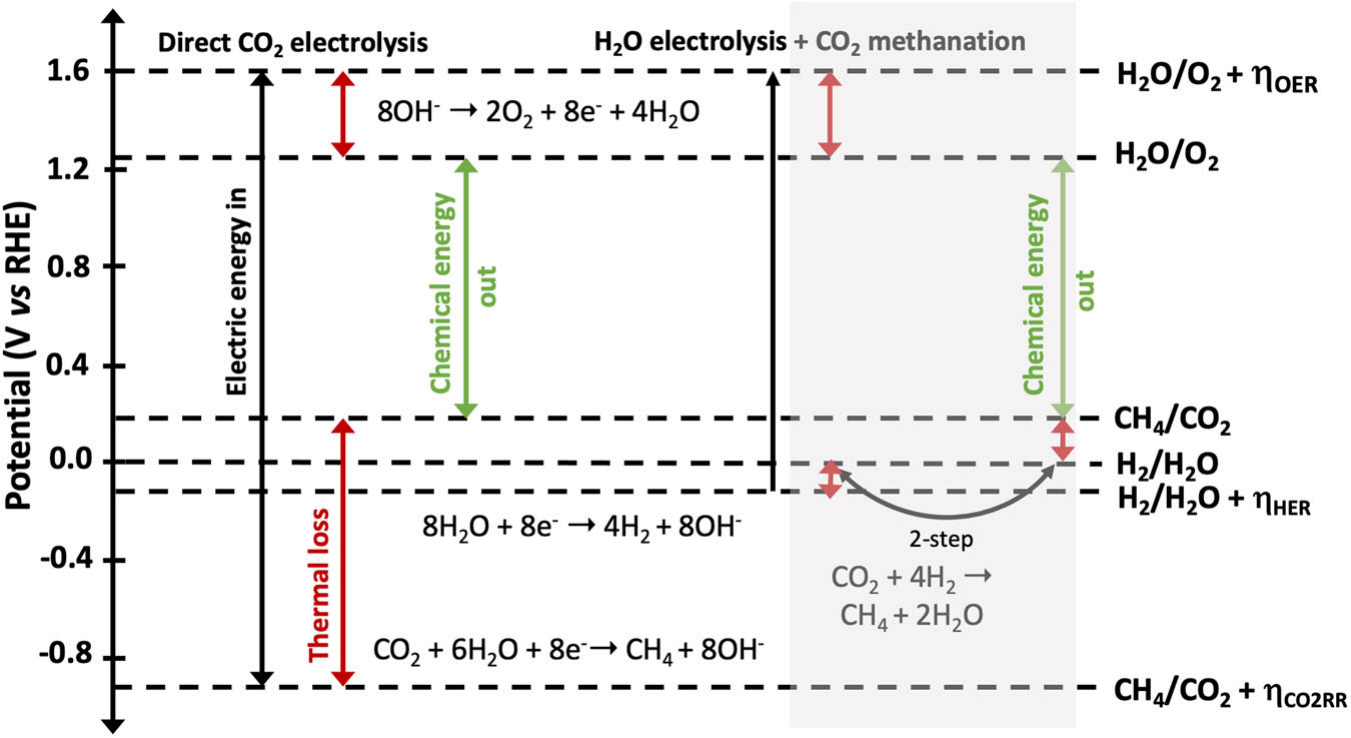
Energy scheme showing the energy inputs and outputs for direct CO_2_ electrolysis and H_2_O electrolysis combined with CO_2_ methanation. Energy lost as heat is marked in red. Redrawn from Sheehan (2021)^59^.

Protons and electrons are usually provided by the water oxidation reaction, but they could also come from the oxidation of organic waste generated by other life support processes. When protons and electrons are provided by the water oxidation reaction, the thermodynamics are similar to the hydrogen evolution reaction and the net reaction requires 1.06 V to proceed at 25 °C, corresponding to 5.16 MWh per ton of CO_2_ reacted^59^. Besides the possibility of operating at lower temperatures and pressures than the currently utilized Sabatier reactor for the thermocatalytic conversion of CO_2_ to CH_4_ and H_2_ on the ISS, the electrosynthesis of CH_4_ from CO_2_ provides the possibility of designing devices with decreased weight. This could lead to a substantial reduction in payload mass, compensating for the lower productivity. Figure 6 depicts an energy diagram comparing the energetic losses associated with direct CO_2_ electrolysis and the coupling of H_2_O electrolysis and CO_2_ methanation as currently present on the ISS. Major energy losses are still observable with the direct CO_2_ electrolysis set-up due to the high overpotential of the electrochemical CO_2_ reduction reaction (CO_2_RR) at the cathode, illustrating that further research into electrocatalysts and electrolyte optimization is required to optimize this system and improve its feasibility for space applications. The power-generating component for an electrosynthesis device could be ultra-light-weight photovoltaic (PV) modules or integrated photoabsorber-electrocatalyst systems, so-called photoelectrochemical (PEC) devices^60–62^. The latter systems provide the possibility of integrating light-absorption, charge separation and catalysis and could represent significant weight and volume advantages. Moreover, in comparison to terrestrial applications of these PEC systems, the entire solar spectrum (AM 0) can be utilized in space. This opens the possibility of using different materials than currently investigated for Earth applications. One possibility is also to utilize non-aqueous electrolytes for the reaction, given the difficulty, complexity and risk associated with extracting water from resources on Mars^60^. This also provides an interesting possibility for generating O_2_ for life support and as a propellant oxidizer as well as a low-grade fuel (CO) in situations when maintaining and recycling water is difficult. This system could also only require sunlight and atmospheric CO_2_ as inputs: the cathodic reaction involves the dimerization and disproportionation of CO_2_ in the presence of a suitable catalyst to yield CO and carbonate anion (CO_3_^2^^−^); the CO is collected and stored for potential use as a fuel, while CO_3_^2−^ is transported across the membrane where it is oxidized to O_2_ and CO_2_^57^:

6 $${{{\mathrm{Cathode}}:4{\mathrm{CO}}}}_{2} + 4{{{\mathrm{e}}}}^{ - } \to 2 {\mathrm{CO}} + 2{\mathrm{CO}}_{3}^{2 - }$$

7 $${{{\mathrm{Anode}}:2{\mathrm{CO}}}}_{3}^{2 - } \to {{{\mathrm{O}}}}_{2} + 2{\mathrm{CO}}_{2} + 4{{{\mathrm{e}}}}^{ - }$$

8 $${{{\mathrm{Overall}}}}\,{{{\mathrm{cell}}}}\,{{{\mathrm{reaction:2CO}}}}_{{{\mathrm{2}}}} \to {{{\mathrm{2CO + O}}}}_{{{\mathrm{2}}}}$$

Molecular catalysts such as Mn(mesbpy)(CO)_3_Cl have been investigated for the reaction terrestrially and several elements of this reaction have been shown to work^60^, but so far, they have not been investigated under conditions relevant to Mars application (lower total and CO_2_ pressure than on Earth as well as lower temperatures). Furthermore, the lower gravitational environment with the lower buoyant force could lead to complications regarding the interaction of gaseous CO_2_ with the electrode surface. Further investigations of (photo-)electrochemical CO_2_ reduction reactions in a simulated Martian environment are therefore required before predictions about applications and device architectures can be made. This includes studies of the catalytic cycle as well as catalyst selectivity.

Hydrogen is an attractive, alternative energy source and an interesting fuel for space applications. Its high-pressure storage and transportation are however challenging. To overcome these issues, storage and transportation of hydrogen can be simplified through chemical transformation to another compound and back again. Ammonia is an attractive and sustainable choice for hydrogen storage (as discussed in the ammonia-based fuel cell section above) as it contains 17.75 wt% hydrogen and does not involve carbon and carbon monoxide species in its synthesis process^32^. In addition, ammonia is found in the liquid state at lower pressures and higher temperatures than hydrogen; thus, it can be stored and transported under less extreme conditions. Much research has been conducted in the area of SOFCs for ammonia oxidation, which crack the molecule at elevated temperatures ranging from 500 and 1000 °C. Currently, the most prominent SOFC for the reaction is the so-called ammonia-fed SOFC-H, an SOFC using protons in the electrolyte as charge carriers^63^. Ammonia is fed into the cell via the anode side, where it decomposes to hydrogen and nitrogen. The formed hydrogen is oxidized to protons, reacting with oxygen to produce water:

9 $${{{\mathrm{Anode}}:2{\mathrm{H}}}}_{2} \to 4{{{\mathrm{H}}}}^{ + } + 4{{{\mathrm{e}}}}^{ - }$$

10 $${{{\mathrm{Cathode}}:{\mathrm{O}}}}_{2} + 4{{{\mathrm{H}}}}^{ + } + 4{{{\mathrm{e}}}}^{ - } \to 2{{{\mathrm{H}}}}_{2}{{{\mathrm{O}}}}$$

The SOFC-Hs remain good ionic conductivity at lower temperatures (see section fuel cells) and also circumvent the formation of NO_x_ species^63^ which are a by-product in SOFCs using O^2−^ ions as charge carriers. SOFCs have also been considered for wastewater treatment, as ammonia, once extracted from wastewater, could be used as a fuel in SOFCs^63^. This is could potentially be very interesting for space applications, where recycling and waste management remain significant challenges for long-term voyages and habitats. Reversible operating ammonia systems (i.e., ammonia-fuel cells combined with ammonia synthesis devices) would also be highly interesting for space applications as the synthesized ammonia could be further on used in the synthesis process of fertilizers. Ammonia waste, on the other hand, could be recycled directly. Such (photo-)electrochemical systems would require however the optimization of stable, efficient catalysts for both reactions, the elimination of the hydrogen evolution reaction during ammonia synthesis and a general optimization of the kinetics and thermodynamics intersection of both reactions.

## Conclusion and outlook

Electrochemistry and electrochemical system engineering will play a key role in future human space exploration^64–69^. Besides their vital importance in O_2_ generation and CO_2_ reduction in life support systems, they can be used in various power and energy storage applications outlined here. Moreover, electrochemistry finds applications in the treatment of raw materials and/or electro-reforming and electro-winning, materials and interface tailoring, material purification such as cadmium or gold, the production of photoabsorbers such as Si, ZnO and CdTe for application in PV or PEC cells, the synthesis of valuable chemicals such as urea and other fertilizers as well as wastewater treatment. Further experiments investigating the fundamental properties of electrochemical energy conversion devices in lunar and Martian environments comprising reduced gravitation and lower temperatures and pressures are however required to understand and optimize key governing processes. As human space exploration faces similar challenges to the green energy transition on Earth, insights gained in these experiments will be crucial as well to understand the importance of natural convection processes on product selectivity and device performance in terrestrial device applications.

### Reporting summary

Further information on research design is available in the Nature Research Reporting Summary linked to this article.

## Supplementary information

Reporting Summary Checklist

## Data Availability

All data and their resources used in this article are available from the authors upon request.
